# Amyand’s hernia with appendicitis masquerading as Fournier’s gangrene: a case report and review of the literature

**DOI:** 10.1186/s13256-016-1046-9

**Published:** 2016-09-22

**Authors:** Kishore Rajaguru, Daniel Tan Ee Lee

**Affiliations:** Department of General Surgery, Ng Teng Fong General Hospital, 1 Jurong East Street 21, Singapore, 609606 Singapore

**Keywords:** Amyand’s hernia, Fournier’s gangrene, Necrotizing fasciitis, Perforated appendicitis

## Abstract

**Background:**

The incarceration of an appendix within an inguinal hernia sac is known as Amyand’s hernia. Appendicitis in Amyand’s hernia accounts for 0.1 % of the cases. An aggressive necrotizing infection of the genitalia and perineum, called Fournier’s gangrene, can rapidly progress to sepsis and death. We describe a rare case of Fournier’s gangrene complicating Amyand’s inguinal hernia which has rarely been reported in the literature.

**Case presentation:**

This case report describes the presentation and management of a 47-year-old Chinese man who presented with pus discharge from his right inguinoscrotal region and lower abdominal pain with clinical signs of Fournier’s gangrene. On surgical exploration, a complicated Amyand’s hernia (Losanoff and Basson classification type 4) was found to be the cause of his Fournier’s gangrene.

**Conclusions:**

A perforated appendix within an inguinal hernia causing Fournier’s gangrene is rarely seen in clinical practice. The diagnosis of this condition is almost always made intraoperatively. Early recognition and awareness of perforated appendicitis within an inguinal hernia sac as one of the causes of Fournier’s gangrene and good surgical technique in such cases are the keys to success when dealing with this surgical issue. In complicated presentations of Amyand’s hernia, an appendicectomy with anatomical repair is the best treatment. It is better to avoid meshplasty.

## Background

The incarceration of a non-inflamed appendix, inflamed appendix, or perforated appendix within an inguinal hernia is termed Amyand’s hernia [1]. The incidence of appendicitis within an inguinal hernia is estimated at 0.07 to 0.13 %. Preoperative diagnosis of appendicitis in obstructed hernia is difficult and almost always the diagnosis is made intraoperatively. Fournier’s gangrene is a rapidly progressive necrotizing infection of the perineum and the genitalia with occasional extension to the anterior abdominal wall. It is a surgical emergency characterized by a synergistic necrotizing fasciitis and the infection usually spreads along the subcutaneous and fascial planes, although myonecrosis is rare. A perforated appendix in an inguinal hernia presenting as Fournier’s gangrene is rarely seen in clinical practice; we encountered such a case in our practice and it demands reporting.

## Case presentation

A 47-year-old mentally impaired Chinese man presented with 4 days’ history of lower abdominal pain and pus discharge from his right inguinoscrotal area. He had had continuous fever with chills and rigors for the past 2 days. He denied any history of diarrhea, vomiting, or abdomen distension. His past medical history included hyperthyroidism and hypertension and he has been on antithyroid medications and beta blockers for more than 20 years.

**Fig. 1:**
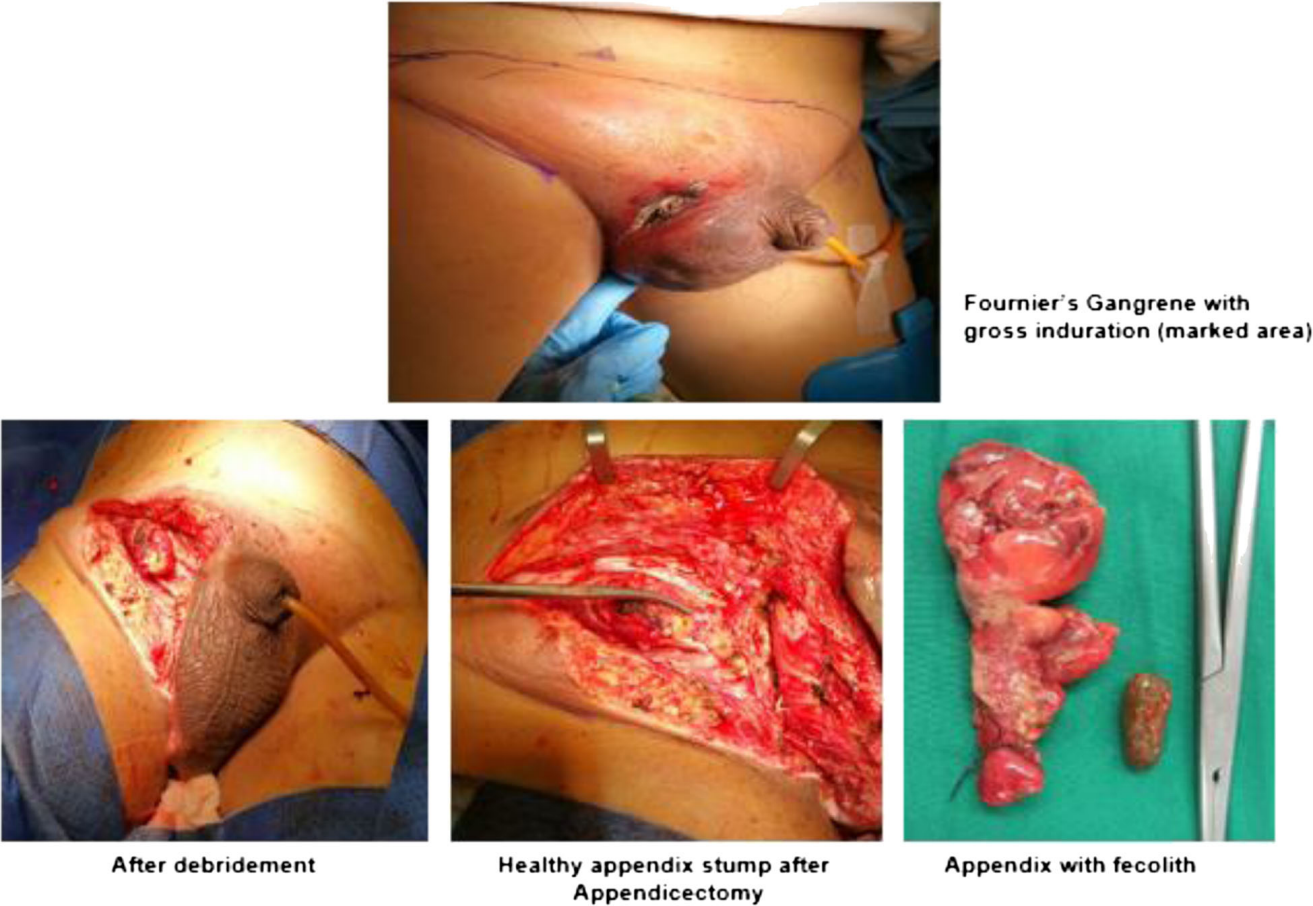
Initial wound debridement and appendicectomy

On examination he was lethargic, toxic, and clinically dehydrated. Initial vital signs showed that he was hypothermic (30.4 °C), hypotensive (74/50 mmHg), with bradycardia (48/minute) and tachypnea (30/minute). Cardiovascular and respiratory examinations were insignificant. His abdomen was soft with tenderness over his lower abdomen; however, his bowel sounds were active. An examination of his groin and external genitalia showed gross necrotizing fasciitis with the epicenter of an abscess at his right groin extending up to the root of his right scrotum (Fig. 1). His right testis was not palpable separately. His left groin, scrotum, and left testis were normal.

Blood investigation revealed hemoglobin (12.5 gm/dL), leucocytosis (33,000 cells/mm^3^), neutrophils 24×10^9^/L, lymphocytes 7.2×10^9^/L, and C-reactive protein (CRP) 320 mg/dL. A renal panel showed signs of acute kidney injury: creatinine 353 μmol/L and estimated glomerular filtration rate (eGFR) 29 mL/min/1.73m^2^. His total bilirubin was 0.6 mg/dl and his albumin was low (22 mg/dl). No significant pathology was found on an X-ray of his chest and abdomen. Blood gas analysis showed metabolic acidosis with lactate 7.7 mmol/l. A thyroid function test revealed undetectable thyroid stimulating hormone (TSH) <0.01 with grossly elevated free thyroxine (T_4_; 59.1 pmol/L). His thyroid peroxidase antibodies (TPOAb) and TSH antibodies were elevated. He was intubated and transferred to our High Dependency (HD) unit for resuscitation and active monitoring.

In view of suspected thyroid crisis (Burch and Wartofsky score of 50) we started him on propylthiouracil and Lugol’s iodine, hydrocortisone was administered intravenously, and he continued to be treated with beta blockers. Broad spectrum antibiotics (penicillin, ceftriaxone, and metronidazole) were started. His blood gases were normalized and his serum lactate dropped to 2.2 mmol/l after a few hours of treatment. In view of the gross sepsis which had triggered the thyroid storm, emergency wound debridement was planned.

On exploration, the entire skin and subcutaneous part covering his right inguinal region, groin, and scrotum were gangrenous (15×12 cm) and these areas were excised. The depth of the necrotic patch extended approximately 5 cm from his skin surface. His right spermatic cord and atrophic testis were gangrenous with slough; hence, a right orchiectomy with excision of the cord structures was done. Debridement of tissues in his right inguinal area showed a thickened indirect hernia sac with gangrenous appendix with pus and a 1 cm fecolith close to the base as the content. His deep inguinal ring was narrowed (0.5 cm). The sac was opened at the fundus. His appendix base was found to be healthy and an appendicectomy was done (Fig. 1). Definitive hernia repair was not attempted in view of extensive infected tissues and his poor physiological status.

**Fig. 2:**
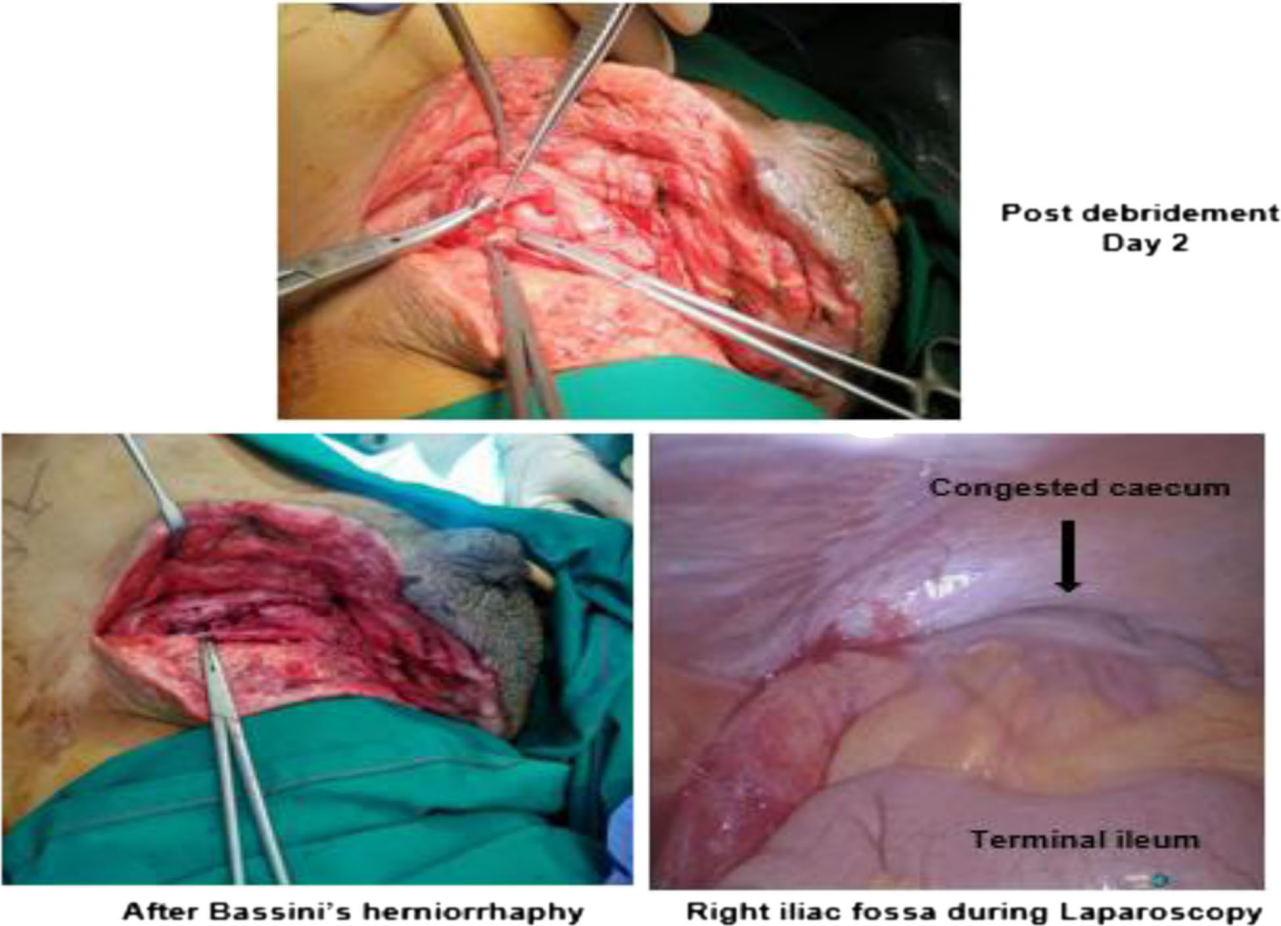
Bassini’s herniorrhaphy

He was extubated and transferred back to our High dependency unit for further monitoring. Post-procedure his inflammatory markers normalized: white blood cells were 9700 cells/mm^3^ and CRP was 54 mg/dL. He made an uneventful postoperative recovery; he received a definitive inguinal hernia repair and diagnostic laparoscopy 48 hours after the debridement to check for the viability of his intestines and to rule out another abdominal source of sepsis. The laparoscopic examination showed a healthy terminal ileum, ascending colon, and congested caecum. Subsequently, the hernial sac with his appendix stump was reduced into his peritoneal cavity. The neck of the sac was transfixed followed by right inguinal modified Bassini’s herniorrhaphy (Fig. 2). A corrugated rubber drain was kept in his inguinal region and the wound was closed partially. He recovered well and the drain was removed on the second day. Secondary skin suturing was done after 3 weeks. He was discharged home with negative pressure wound dressing. An ultrasound of his neck was done, which showed diffusely enlarged thyroid gland with increased vascularity but no nodules; this finding supports a diagnosis of Grave’s disease. Currently he is in good health with no evidence of hernia recurrence. His TSH and free T_4_ levels are within normal limits; he is continuing with antithyroid medications with plans for radioactive iodine ablation at a later date.

## Discussion

The incidence of Amyand’s hernia is between 0.4 and 0.6 %, which is smaller than the classical incidence of 1 % which was based on older research [2]. The association of appendicitis is even rarer and reported to be 0.07 to 0.13 % [2]. The majority of reported cases of Amyand’s hernia are right sided, which can be explained by the anatomical location of the organ and by the fact that right-sided inguinal hernias are more common than left-sided inguinal hernias. Few cases of left-sided Amyand’s hernia have been reported in the literature. An excessively long appendix, mobile caecum, situs inversus, and intestinal malrotation may be possible reasons for a left-side presentation [3].

Acute appendicitis in Amyand’s hernia is usually caused by extraluminal obstruction due to pressure on the hernia neck by a narrow deep inguinal ring or from an intraluminal obstruction caused by a fecolith, although the former is more common [4]. An appendix will be retained after entering the hernia sac by formation of adhesions; inflammation of the appendix results from changes in intra-abdominal pressure and frequent contraction of the abdominal wall muscles, which causes strangulation of the appendix. Translocation of virulent bacteria through the hernia sac into subcutaneous and cutaneous tissues promotes the rapid spread of disease causing localized ischemia. The compromised immune status of the individual provides a favorable environment for rapid multiplication and spread of infection. Ultimately, all these events lead to obliterative endarteritis and thrombosis of subcutaneous vessels which result in Fournier’s gangrene.

Amyand’s hernia usually presents with a painful inguinoscrotal mass [5] and a majority of cases are misdiagnosed as strangulated or incarcerated inguinal hernia. Richter’s hernia, testicular torsion, testicular tumor with hemorrhage, inguinal adenitis, epididymitis, and orchitis also present with features similar to those of Amyand’s hernia. Amyand’s hernia can be diagnosed preoperatively by ultrasound or computed tomography (CT) scans [6], but in most cases the diagnosis was made intraoperatively.

A simplified classification of Amyand’s hernia was defined by Fernando and Leelaratna in which they described an inguinal hernia containing: (a) a non-inflamed appendix, (b) an inflamed appendix, or (c) a perforated appendix [7]. The surgical management of Amyand’s hernia depends on the status of the appendix; a formal classification (Table 1) was proposed by Losanoff and Basson [8].

**Table 1:** Losanoff and Basson's classification of Amyand's hernia

Losanoff and Basson classification [8]	Description	Surgical management
Type 1	Normal appendix within an inguinal hernia	Hernia reduction, mesh repair, appendicectomy only in young patients
Type 2	Acute appendicitis within an inguinal hernia, no abdominal sepsis	Appendicectomy through hernia, primary repair of hernia, no mesh
Type 3	Acute appendicitis within an inguinal hernia, abdominal wall, or peritoneal sepsis	Laparotomy, appendicectomy, primary repair of hernia, no mesh
Type 4	Acute appendicitis within an inguinal hernia, related or unrelated abdominal pathology	Manage as types 1 to 3 hernia, investigate or treat second pathology as appropriate

Future appendicitis in left-sided Amyand’s hernia may cause a diagnostic dilemma due to the unusual position of the appendix. Johari* et al.* suggested routine appendicectomy in all left-sided Amyand’s hernias irrespective of the presence of an inflamed appendix or not [9]. Mesh repair of the hernias should be avoided in the presence of an infected field due to higher chances of mesh infection and hernia recurrence.

Type 4 Amyand’s hernia with Fournier’s gangrene as the second pathology was found in our case. Broad spectrum antibiotics covering Gram-positive and Gram-negative bacteria and anaerobes should be started when clinical diagnosis of Fournier’s gangrene is made. Aggressive surgical debridement of devitalized tissues is the key factor, since delaying surgical treatment leads to higher mortality rates. On intraoperative examination, extensive gangrenous tissues will be seen in the peritesticular tissues which mimic gangrene of the testis leading to orchidectomy, although a pathological review shows they are not involved [10]. A possible intra-abdominal source of infection is suspected in the presence of testicular involvement. Urinary or fecal diversion may be necessary to prevent wound contamination. Maintaining a positive nitrogen balance with adequate nutrition and local negative pressure dressing proved to have a good impact on the management of wounds [11]. The use of hyperbaric oxygen therapy is still a topic of debate in wound management. More frequently these wounds require reconstructive surgery in the form of split skin grafting and flap reconstructions.

## Conclusions

The incarceration of an appendix within an inguinal hernia sac is known as Amyand’s hernia. An aggressive necrotizing infection of the genitalia and perineum, called Fournier’s gangrene, can rapidly progress to sepsis and death. Fournier’s gangrene complicating Amyand’s inguinal hernia has rarely been reported in the literature. Delay in surgical intervention contributes to increased morbidity and mortality. This case report explains the features, pathophysiology, and the importance of surgical intervention in this rare condition and it improves awareness of the presence of perforated appendicitis within an inguinal hernia sac, which is one of the rarer causes of Fournier’s gangrene. Our patient met the criteria for Losanoff and Basson’s type 4 Amyand’s hernia. It is better to avoid meshplasty in the presence of pus or perforated appendix. Early recognition of Fournier’s gangrene and aggressive operative debridement are essential to ensure the best possible outcome.
